# Drought stimulates root exudation of organic nitrogen in cotton (*Gossypium hirsutem*)

**DOI:** 10.3389/fpls.2024.1431004

**Published:** 2024-11-19

**Authors:** Harrison R. Coker, Heng-An Lin, Caleb E. B. Shackelford, Malak M. Tfaily, A. Peyton Smith, Julie A. Howe

**Affiliations:** ^1^ Department of Soil and Crop Sciences, Texas A&M University and Texas A&M AgriLife, College Station, TX, United States; ^2^ Department of Environmental Sciences, University of Arizona, Tucson, AZ, United States

**Keywords:** root exudates, drought, nitrogen, metabolomics, cotton, FT-ICR-MS

## Abstract

Root exudation of N is a plant input to the soil environment and may be differentially regulated by the plant during drought. Organic N released by root systems has important implications in rhizosphere biogeochemical cycling considering the intimate coupling of C and N dynamics by microbial communities. Besides amino acids, diverse molecules exuded by root systems constitute a significant fraction of root exudate organic N but have yet to receive a metabolomic and quantitative investigation during drought. To observe root exudation of N during drought, mature cotton plants received progressive drought and recovery treatments in an aeroponic system throughout their reproductive stage and were compared to control plants receiving full irrigation. Root exudates were nondestructively sampled from the same plants at 9 timepoints over 18 days. Total organic C and N were quantified by combustion, inorganic N with spectrophotometric methods, free amino acids by high performance liquid chromatography (HPLC), and untargeted metabolomics by Fourier-transform ion cyclotron resonance-mass spectrometry (FT-ICR-MS). Results indicate that organic N molecules in root exudates were by far the greatest component of root exudate total N, which accounted for 20-30% of root exudate mass. Drought increased root exudation of organic N (62%), organic C (6%), and free amino acid-N (562%), yet free amino acids were <5% of the N balance. Drought stress significantly increased root exudation of serine, aspartic acid, asparagine, glutamic acid, tryptophan, glutamine, phenylalanine, and lysine compared to the control. There was a total of 3,985 molecules detected across root exudate samples, of which 41% contained N in their molecular formula. There were additionally 349 N-containing molecules unique to drought treatment and 172 unique to control. Drought increased the relative abundance and redistributed the molecular weights of low molecular weight N-containing molecules. Time-series analysis revealed root exudation of organic N was stimulated by drought and was sensitive to the degree of drought stress.

## Introduction

Root exudates stimulate the growth and diversity of microbial populations and signal the recruitment of beneficial microorganisms (Bais et al., 2006; Canarini et al., 2019). In turn, soil microorganisms influence the plant metabolome (Badri et al., 2013) and assist plants in enduring biotic and abiotic stresses (Zolla et al., 2013). The molecular profile of root exudates is sensitive to abiotic factors (Chai and Schachtman, 2022) and changes with plant growth stage (Ofosu-Budu et al., 1990; Tixier et al., 2023), day-night cycles (Ofosu-Budu et al., 1990; Tixier et al., 2023), and disturbances such as nutrient stress (Carvalhais et al., 2011). Furthermore, the composition and quantity of root exudates respond to drought severity and recovery (Lin et al., 2023), which differs by species and genotype (Canarini et al., 2016; Gargallo-Garriga et al., 2018; Williams and de Vries, 2020; Chen et al., 2022). Understanding the effects of drought on root exudation behavior can be used to harness drought-resilient rhizosphere microbiomes for improved climate resilience in agronomic crops that are increasingly threatened by water-limited environments (de Vries et al., 2020). Further, the effect of drought and subsequent recovery on root exudation mechanisms in agriculturally relevant crops requires significant attention (Williams and de Vries, 2020).

Throughout the growth period of wheat (*Triticum aestivum*), root exudation of N can account for up to 19% of total N in low-N plants and up to 33% in high-N plants (Janzen, 1990). The release of organic N from root systems may be a plant mechanism to balance internal N concentrations, as wheat (*Triticum aestivum*) possesses the ability to “salvage” up to 90% of lower molecular weight amino acids (Warren, 2015). Root exudation of inorganic N forms such as ammonium (NH_4_^+^) and nitrate (NO_3_^-^) also occurs (Feng et al., 1994; Jones et al., 2009; Scheurwater et al., 1999). Many plants stimulate organic N exudates, such as amino acids, organic acids, and sugars, in response to nutrient deficiency as a strategy to cope with limiting abiotic conditions. This has been observed with N, P, K, and Fe deficiency in sorghum (*Sorghum bicolor;* Carvalhais et al., 2011), K deficiency in maize (*Zea mays*; Kraffczyk et al., 1984), Zn deficiency in grasses (Zhang et al., 1991), and P deficiency in legumes (Lipton et al., 1987).

Root exudates have been considered a largely N-free milieu apart from amino acids (Moe, 2013). Rhizosphere microorganisms use amino acids to fulfill their C requirements, particularly when C is limiting for microbial use (Farrell et al., 2014). Amino acid efflux from root systems was previously assumed to occur as passive diffusion at the root tip (Jones and Darrah, 1994). However, recent evidence suggests plant trait-based controls on the concentration and profile of amino acids in root exudates (Lesuffleur and Cliquet, 2010; Kanté et al., 2023). Further, microbial exudation in the rhizosphere may also control amino acid efflux in plant roots (Phillips et al., 2004). Free amino acids are a primary system of osmolyte (i.e., compatible solute) movement during water-stress (Burg and Ferraris, 2008), thus increased accumulations of free amino acids in root tissues and root exudates is an expected effect of drought. Among temperate legumes (e.g., alfalfa; *Medicago sativa*), amino acids are the most prevalent class of low molecular weight N-containing molecules in root exudates (Paynel et al., 2008). However, the extent to which free amino acids contribute to the N balance of root exudates remains unclear. For instance, Waldo et al. (2021) quantified amino acids as less than half of organic N entering the rhizosphere of a bog environment. However, root exudation of N extends beyond amino acids; in reality, root exudates are a complex mixture of molecules resultant of plant metabolism along with nutrients previously taken up bound in organic molecules (Rengel et al., 2022).

While microbial biomass is C-limited in the bulk soil, N is the limiting nutrient in the rhizosphere (Wardle, 1992; Rengel et al., 2022). Low molecular weight C- and N-containing molecules in root exudates are also assimilated by microorganisms to synthesize proteins and other N-containing organic molecules (Haichar et al., 2008; Grzyb et al., 2021). The exudation of higher weight (>500 Da) N and S molecules are tied to microbial priming of oligotrophic environments, with a greater abundance of N-containing molecules promoting greater microbial activity when C supplies are limited (Waldo et al., 2021). To provide context to this finding, the degradation of high molecular weight (HMW) proteins into low molecular weight (LMW) peptides and amino acids is hypothesized to be at the frontier of plant-microbial competition for N in soils (Farrell et al., 2014). Nonetheless, plants utilize contrasting economic strategies to regulate C and N cycling in continuous feedback with rhizosphere microorganisms (Henneron et al., 2020a, b; Coskun et al., 2017), such as in a study of three tropical rainforest trees where drought tolerance strategies of root exudation were strategically altered in a species-specific manner (Honeker et al., 2022).

To date, total N content of root exudates during drought has been limited to an environmental observation from a temperate steppe environment that found reduced availability of soil water and N increased total N exudation and decreased the C:N ratio of root exudates (Li et al., 2021), along with an experimental study that shows tomato increased root exudation of organic N during drought (Preece et al., 2023). Many studies have clarified that drought alters the concentration of amino acids in root exudates (Hildebrand et al., 2023; Honeker et al., 2022; Gargallo-Garriga et al., 2018; Lesuffleur et al., 2007; Shaposhnikov et al., 2016; Jamil et al., 2018; Calvo et al., 2017). On the microbial side, increased exudation of N-containing molecules such as enzymes (e.g., protease and catalase) have been linked to drought tolerance in maize (*Zea mays*) (Song et al., 2012).

Recent untargeted metabolomics using Fourier transform ion cyclotron resonance mass spectrometry (FT-ICR-MS) have revealed the vast and largely uncharted molecular profile of root system efflux. Root exudation of peas (*Pinus sativum*) was characterized by a significant number of N-containing molecules in a wide range of nucleosides, cytokines, amino acids, small oligopeptides, fatty acid amides, alkaloids, and phosphocholines (Calabrese et al., 2023). In a study of six plant species, degree of unsaturation and N content were the two key properties useful to distinguish the similarities and differences between root exudation of various species (Miao et al., 2020). In maize, Lohse et al. (2022) reported that greater root exudation rates correlated to greater presence of organic N-containing molecules. They further hypothesized that organic N in root exudates (e.g., growth factors, vitamins, fatty acids, and other secondary molecules) may be responsible for a large portion of high molecular diversity, although being exuded in smaller quantities than other C-containing compounds.

Root exudation of organic N, a principal element of plant and microbial nutrition, has received limited investigation during drought. Using cotton as an agronomically important model crop, the central hypothesis of this study is that drought stress alters root exudation of N as a mechanism of drought tolerance similar to altered C exudation. By pairing quantitative and nontargeted techniques, the extent that organic N and amino acids contribute to total N of root exudates, and how the N metabolome qualitatively shifts as a result of drought, were investigated. The objectives were to 1) quantify organic N exudation from whole root systems non-destructively and 2) identify drought-induced shifts in the N metabolome of root exudates by comparing root exudates of drought-treated plants to well-watered control plants during progressive drought stress and subsequent recovery in a time-series experiment.

## Materials and methods

### Experimental design and sample collection

Cotton (*Gossypium hirsutum* cv. TAM 421*)* was used as the model crop as it is frequently grown with no or deficit irrigation and its yield is known to be threatened by drought and heat stress (Ul-Allah et al., 2021). Plants were grown to the reproductive physiological stage (match-head square stage; Oosterhuis, 2015) in a growth chamber using the free-draining high-pressure aeroponic system described in Lin et al. (2022), using three plants per pot and five replications of each treatment. In brief, nutrient solution was sprayed directly onto plant roots. The treatment phase (11 days) consisted of two treatments: progressive drought and well-watered control. Progressive drought was applied by gradually reducing irrigation provided to root systems every 2-3 days to mimic drying of the soil environment, which was accomplished by increasing the interval between irrigation events as DAT progressed (**Table 1**). At the end of the treatment phase, when plants exhibited extreme drought stress, full irrigation was applied to all plants during the recovery phase (7 days) until plants no longer expressed visual drought symptoms. Nutrients were delivered in the form of full-strength Hoagland’s solution (Hoagland and Arnon, 1950).

**Table 1:** The aeroponic irrigation schedule used to apply progressive drought and drought recovery.

DAT	Irrigation rate	On	Off		Irrigation Provided		Irrigation Provided
	mL min^-1^	sec	min		mL h^-1^		% control
			Control	Drought	Control	Drought	Drought
0	65.4	0:10	5:00	5:00	130	130	100%
2	65.4	0:10	5:00	10:00	130	65	50%
4	65.4	0:10	5:00	20:00	130	33	25%
7	65.4	0:10	5:00	30:00	130	22	17%
9	65.4	0:10	5:00	40:00	130	16	13%
11	65.4	0:10	5:00	50:00	130	13	10%
14	65.4	0:10	5:00	5:00	130	130	100%
16	65.4	0:10	5:00	5:00	130	130	100%
18	65.4	0:10	5:00	5:00	130	130	100%

**Figure 1:**
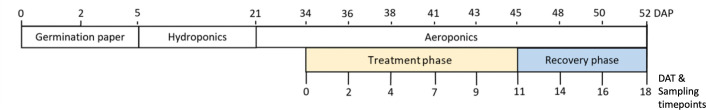
Timeline of experiment. Plants were germinated and transferred to hydroponics 5 days after planting (DAP). At 21 DAP, plants were transferred from hydroponics to aeroponics. The treatment phase began at the match-head stage of cotton on 0 days after treatment (DAT). During the treatment phase, drought and well-watered (control) treatments were applied. A 7-day recovery phase, where all plants received well-watered conditions, followed the 11-day treatment phase.

Root exudates were sampled by replacing the nutrient solution with a calcium chloride sampling solution (0.05 mM CaCl_2_, pH 6.0-6.5). The use of CaCl_2_ increases the ionic strength of the sampling solution to be more representative of the soil solution, which can range up to 14 mM Ca^2+^ in calcareous soils (Fried and Shapiro, 1961). Whole root systems were rinsed with sampling solution for 2 min (65.4 mL min^-1^) and the root runoff collected, referred to as “rinseate”. Rinseate was collected during the afternoon between 14:00 and 16:00 on collection days. Rinseate was immediately filtered through a 0.22-μm polyethersulfone syringe filter and stored at -80°C. Plants were non-destructively sampled at 0, 2, 4, 7, 9, 11, 14, 16, and 18 days after treatment phase began (**Figure 1**). Five out of ten pots for each treatment were randomly selected for sample collection on each sampling day. A total of 90 samples were collected. At the end of treatment phase (day 11), half of the pots were destructively sampled and weighed for biomass, while the remaining pots were harvested at the end of recovery (day 18). Additional details about the experimental design, aeroponic system for drought treatment and root exudate collection, and plant performance during drought are provided in Lin et al. (2022).

### Quantitative analysis of root exudates

Total C and total N concentrations of root exudates (mg L^-1^) were determined using a TOC analyzer (TOC-L Series, Shimadzu, Japan) at the end of treatment (day 11) and recovery (day 18) phases. Inorganic carbon was considered negligible, thus total carbon was considered equivalent to total organic carbon (TOC). Inorganic N as nitrate-N (NO_3_-N) and ammonium-N (NH_4_-N) were quantified using the spectrophotometric methods described by Doane and Horwáth (2003) and Verdouw et al. (1978). No NH_4_-N was detected; however, NO_3_-N was provided to roots as part of the Hoagland nutrient solution and considered an artifact of plant culture. Thus, NO_3_-N was quantified and subtracted from each total nitrogen measurement to derive total organic nitrogen (TON). Inorganic N represented approximately 20% of total N.

Free amino acids were quantified using high performance liquid chromatography (HPLC) at the Protein Chemistry Lab at Texas A&M University. Samples were dried and concentrated in a vacuum concentrator and resuspended in a sodium borate buffer solution (target pH 8.2) prior to derivatization with o-phthalaldehyde and 9-fluoromethyl-chloroformate. Internal standards (norvaline and sarcosine) were added prior to derivatization. Derivatized amino acids were detected using UV (338/390 nm primaries and 266/324 nm secondaries) spectroscopy and quantified with external standards. For all quantification measurements, two analytical replicates were averaged to represent each experimental unit value.

Quantitative analysis of root exudates (total C, total N, C:N, and NO_3_^-^) was expressed in three different forms: concentration per plant, mass per plant, and specific exudation per plant. Mass per plant (mg) was calculated by multiplying exudate concentration by total rinseate solution provided during exudate collection (i.e., 128.8 mL). Exudate composition was also evaluated on a root dry weight basis to assess the impact of root biomass on exudation magnitude, which will be referred to as specific exudation; this was calculated by multiplying elemental mass exuded per plant by volume of exudate collection and dividing by its respective total dry root biomass. The carbon to nitrogen ratio (C:N) of root exudates was calculated by dividing total C exudation (mg) per plant^-1^ (mg) by total N exudation (mg) per plant^-1^. Amino acids were reported on a concentration basis as measured by HPLC. Because three plants were grown per pot, quantitative results were divided by 3 to represent exudation magnitude of a single plant.

### Nontargeted metabolomics using FT-ICR-MS

Collected root rinseate was processed through solid phase extraction (SPE) (Dittmar et al., 2008) and directly injected into a FT-ICR-MS (12 T Bruker SolariX, USA) for metabolomic analysis at the University of Arizona. One mL aliquots of root rinseate were diluted to 15 mL with sterile deionized water and acidified to pH 2-3 with 150 μL of 1 M HCl. Styrene-divinylbenzene SPE cartridges (Bond Elut PPL, Agilent, Santa Clara, CA) for polar compounds were attached to a glass vacuum manifold and activated with 3 mL methanol under vacuum (16.9 kPa). Samples were added to the cartridge and followed by 30 mL 0.01 M HCl to rinse the cartridge of potential salts. The cartridges were allowed to air dry for 20 min and followed with a final elution of 1.5 mL methanol that gravity drained into 2-mL autosampler vials. Extracted samples were stored at -20°C.

A standard Bruker ESI source was used to generate negatively charged molecular ions and samples were introduced directly to the ESI source. Due to instrument limitation, positive ion mode was not able to be utilized. Scans (144) were averaged for each sample and internally calibrated using an organic matter homologous series separated by 14 Da (CH_2_ groups). Data Analysis software (BrunkerDaltonik version 4.2) was used to convert raw spectra to a list of mass per charge (m/z) values by applying FTMS peak picker module with a signal-to-noise ratio (S/N) threshold set to 7 and absolute intensity threshold to the default value of 100. Putative chemical formulae were then assigned using Formularity (Tolić et al., 2017) software (Tfaily et al., 2018). Chemical formulae were assigned based on the following criteria: S/N > 7 and mass measurement error < 1 mg kg^-1^, taking into consideration the presence of elements C, H, O, N, S and P while excluding other elements (i.e., CHON, CHON-S, CHON-P, CHON-SP). Raw data produced by FT-ICR-MS (peak masses, peak intensities, and metabolic molecular formula) were then processed through the Metabodirect pipeline (Ayala-Ortiz et al., 2023). Peaks (m/z) < 200 and > 900 were filtered using *-m* option, which optimized accurate mass readings and reduced analytical bias at the fringes of FT-ICR-MS scanning range. Data was z-score normalized using *–norm_method* option. The quality control steps, including ^13^C isotope filtering and error filtering (0.5 ppm) followed the default setting. The sampling solution (blanks) used for root exudate collection were also analyzed and detected peaks were removed from further analysis. Peaks identified in less than two samples were also excluded from analysis. Peaks with assigned molecular formula were mapped to Kyoto Encyclopedia of Genes and Genomes (KEGG) database (Kanehisa and Goto, 2000), although there were no KEGG matches for molecules containing N in their molecular formula (N-containing molecules). For a broad overview of KEGG associations observed in all FT-ICR-MS peaks from this dataset, see Lin et al. (2023). **Table 2** includes the workflow of FT-ICR-MS data processing.

**Table 2:** Workflow of FT-ICR-MS data.

Processing steps of FT-ICR-MS data	Counts
Peaks detected	33,870
Peaks after quality control including blank removal	13,033
Molecules assigned molecular formula	3,985
Molecules assigned molecular formula containing N	1,616

Peaks detected were subjected to quality control measures and were then assigned molecular formula.

### Qualitative analysis of N-containing molecules

Molecule peak intensities were transformed to a presence/absence basis. The relative abundance (RA) of N-containing molecules was calculated per sample by dividing the count of N-containing molecules by the count of all molecules and multiplying by 100. The succession of N-containing molecules in the drought-treated samples was assessed using a relative abundance response ratio; the RA of samples was averaged by day and treatment, and drought treatment was normalized to control using Equation 1:


(1) $$relative\;abundance\;response\;ratio=\;\frac{RA_{drought}-RA_{control}}{RA_{drought}}\times 100$$

The proportion of elemental groups (i.e., CHON, CHON-S, CHON-P, CHON-SP, and non-N containing peaks) was calculated per sample by dividing the number of peaks associated with each elemental group over the number of total molecules identified with FT-ICR-MS. The generated m/z values from FT-ICR-MS are equivalent to the calculated molecular masses (amu), which was verified by multiplying the count of each element in a molecule by its molecular weight. For the purposes of this paper, the molecular weights of N-containing molecules were binned into low molecular weight (LMW; 200 – 600 amu) and high molecular weight (HMW; 600 – 900 amu) classes to assess their distributions. Molecular weights were plotted with molecule counts in smoothed (kernel) density plots by treatment and experimental phase, and with a smoothed LOESS regression using 50% spline and 95% confidence interval (CI).

### Statistical analysis

All figures and statistical analyses were generated using R Studio statistical software (v4.2.0 (R Core Team, 2022). Data and code used for this study can be accessed at the following GitHub link: https://github.com/harrisoncoker/Nitrogen-root-exudation-Gossypium-hirsutum. Quantitative measurements of TOC, TON, and free amino acids were tested using bootstrapped two-sample t-tests with Benjamini-Hochburg *p*-value adjustment. Molecular masses were analyzed with two-sample Kolomogrov-Smirnov tests to detect distribution differences between treatments. The FT-IRC-MS dataset of N-containing molecules separated into three distance matrices (baseline, treatment phase, and recovery phase) using Bray-Curtis dissimilarity on a presence or absence basis. Permutational multivariate analysis of variance (PERMANOVA) was performed for each of the three matrices using treatment, day, and the interaction of treatment × day as independent variables. All figures were created using the “*ggplot2”* R package (Wickham, 2016). PERMANOVA was performed in the community ecology “*vegan”* package (Oksanen et al., 2022). The proportions of elemental groups (CHON, CHON-S, CHON-P, CHON-SP, and non-N containing molecules – i.e., “NonN Metabolites”) were used as main factors in a principal components analysis (PCA) with non-numerical experimental metadata (treatment and phase) used as supplemental variables. Squared cosines of main and supplemental factor variables were calculated for all components. Squared cosine values denote the proportion each variable is represented by a specific component. The sum of the squared cosine values across all components is equal to one for each variable. The PCA was performed in JMP Pro 17. Statistical significance was defined as α *<* 0.05.

## Results

### Plant performance during drought

**Figure 2:**
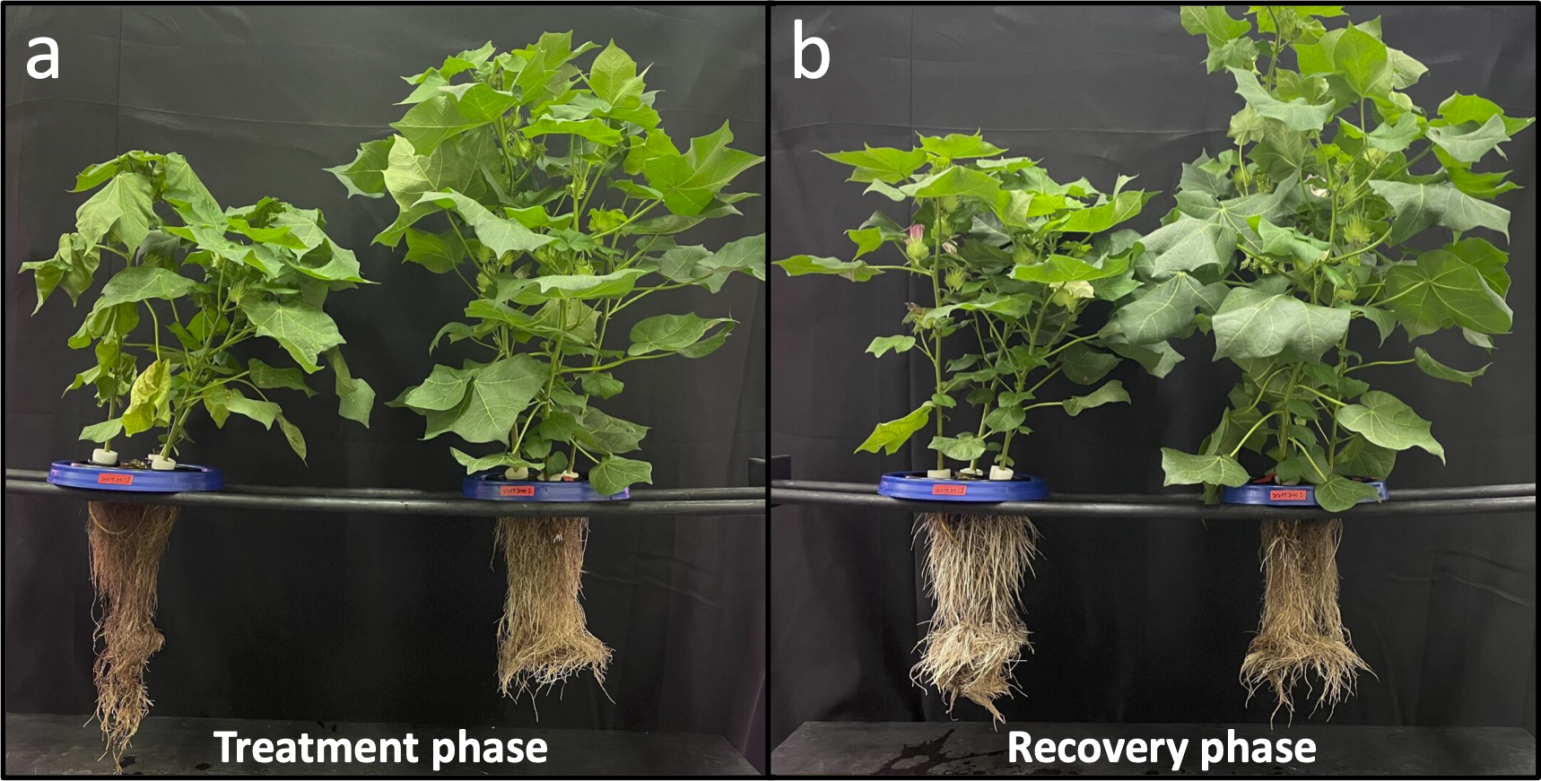
Cotton plants were subjected to progressive drought during the treatment phase **(A)** and subsequent recovery during the recovery phase **(B)** in an aeroponic system. Control plants located on the right of each picture were provided full irrigation throughout the experiment. By the end of treatment phase, drought treatment was exhibiting signs of extreme drought, but had visually fully recovered by the end of the recovery phase. Plants in **(B)** show flowers as the experiment was conducted during the reproductive stage of the plants’ life cycles.

The first signs of visual drought were observed on day 4, with severe drought observed from days 7-9 and extreme drought on day 11. The aboveground biomass of the plants was visually fully recovered after one week of recovery at full irrigation rate (day 18). Plants responded rapidly to progressive drought with reduced plant height, number of green leaves, shoot and root fresh biomass, and shoot dry biomass (**Figure 2**). For a comprehensive analysis of the aeroponic system and plant performance from this study, see Lin et al. (2022) where details of the aeroponic system and plant response to drought are fully covered.

### N balance of root exudates

**Figure 3:**
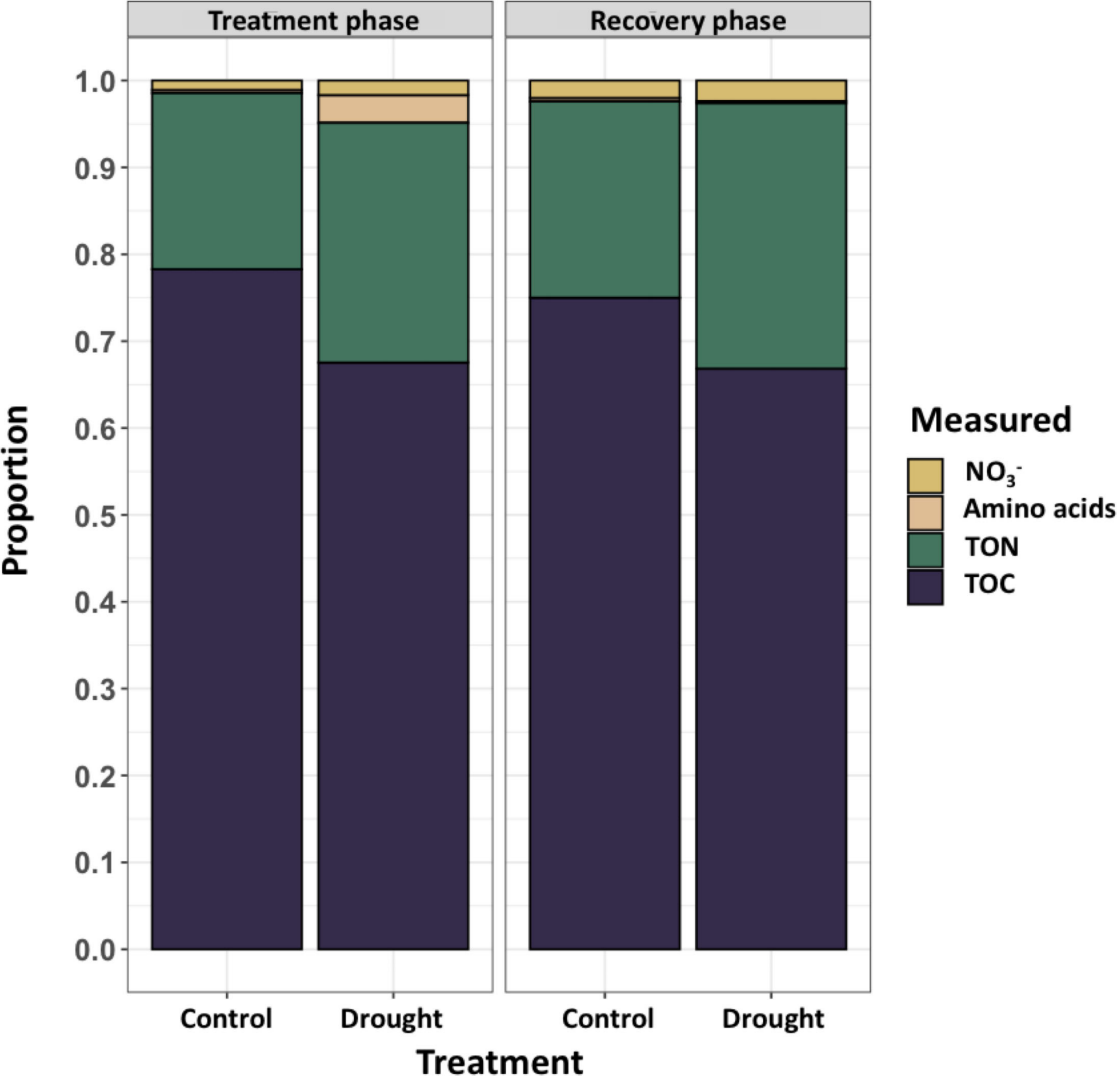
Concentrations (mg L^-1^) of measured constituents in root rinseate samples were quantified at two timepoints, treatment phase (day 11; left) and recovery phase (day 18; right). Rinseate samples were analyzed for total organic carbon (TOC), total organic nitrogen (TON), nitrate (NO_3_^-^) and free amino acids. During treatment phase, drought increased exudation of TOC (*P* = 0.043), TON (*P* = 0.004), and during recovery phase TON was increased (*P* = 0.003) compared to control. Large variation in amino acids led to no differences between treatments. NO_3_^-^ was considered an artifact of the nutrient solution provided by the aeroponic system that was washed from plant roots during root exudate collection, and there were no differences between treatments..

Root exudates from end of drought (day 11) and end of recovery (day 18) were analyzed on a mass balance that consisted primarily of organic carbon (TOC, 65-80%), organic nitrogen (TON, 20-30%), nitrate (NO_3_^-^,< 5%), and free amino acids comprising the remaining components (**Figure 3**). Free amino acids constituted a small portion of rinseate samples except as an effect of drought during the treatment phase (~5%). NO_3_^-^ was likely present as residual nutrient solution on root surfaces and was rinsed from roots while sampling exudates.

The effect of drought during the treatment phase increased TOC by 6% (*P* ≤ 0.001) compared to control, but it did not affect specific TOC (**Table 3**). There was no effect of drought recovery on TOC between treatments. The concentration of TON increased by 62% (*P* ≤ 0.001) and specific TON by 71% (*P* = 0.02) during the treatment phase compared to control. Drought recovery retained a similarly increased TON by 63% (*P* = 0.005) and specific TON by 60% (*P* = 0.002) compared to control. However, TOC did not differ between treatments leading to a drought reduced C:N ratio (*P* ≤ 0.001). Specific exudation of TOC show no differences between treatments at either timepoint, but specific exudation of TON was increased in the drought treatment at both timepoints. Thus, root exudation of C returned to normal conditions within the 7-day recovery period, but root exudation of N remained increased after the cessation of water stress, indicating that a full recovery of root exudation behavior did not occur due to altered release of organic N.

**Table 3:** Root exudate composition reported as concentration, mass, and specific exudation of total organic carbon (TOC), total organic nitrogen (TON), C:N ratio, nitrate (NO_3_^-^), and free amino acids.

Sampling timepoint	Concentration (mg L^-1^)		Mass (mg)		Specific exudation (mg g^-1^ root DW)	
	Control	Drought	Control	Drought	Control	Drought
Treatment						
TOC	**79.0 ± 4.2**	**84.2 ± 2.4**	**10.2 ± 0.5**	**10.8 ± 0.5**	3.4 ± 0.7	3.4 ± 1.1
TON	**20.4 ± 6.8**	**34.4 ± 3.6**	**2.6 ± 0.8**	**4.4 ± 0.5**	**0.8 ± 0.2**	**1.4 ± 0.5**
C:N	**4.2 ± 1.1**	**2.5 ± 0.2**	–	–	–	–
Recovery						
TOC	77.5 ± 0.8	78.5 ± 3.9	10.0 ± 0.1	10.1 ± 0.5	10.1 ± 0.6	10.1 ± 0.7
TON	**23.4 ± 5.2**	**35.8 ± 4.2**	**3.0 ± 0.7**	**4.6 ± 0.5**	**0.6 ± 0.2**	**1.0 ± 0.1**
C:N	**2.1 ± 0.5**	**3.4 ± 0.7**	–	–	–	–

Specific exudation is mass normalized to root dry weight (DW) of the plant. NO_3_^-^ and amino acids mass are of N (NO_3_^–^N and amino acid-N). Data are presented as mean ± standard deviation (SD). Bold values indicate significance (α = 0.05) between treatments at that sampling timepoint.

### Drought-induced changes in the N metabolome

**Figure 4:**
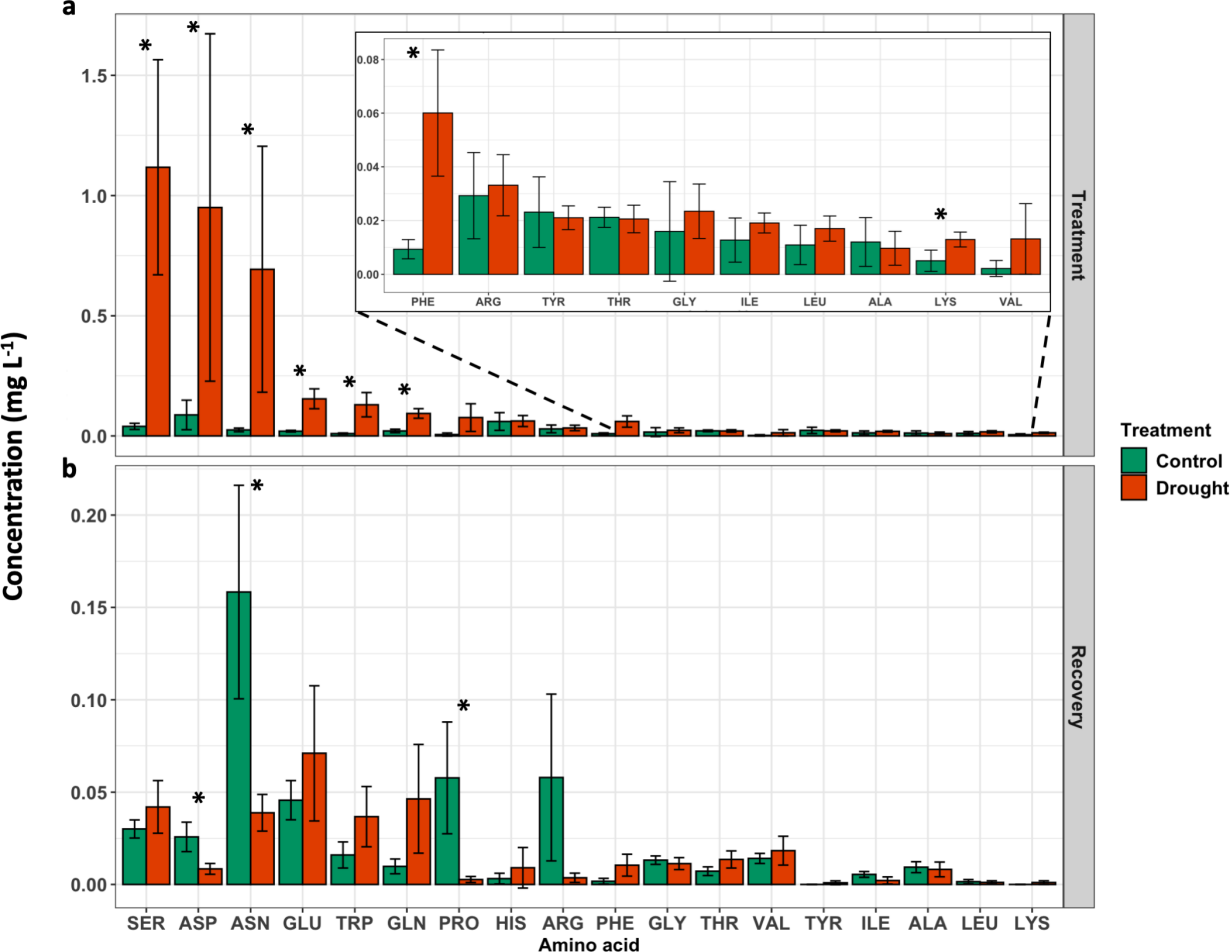
Free amino acid concentration in root exudates quantified at the **(A)** end of treatment phase (DAT 11), and **(B)** end of recovery phase (DAT 18). Results are presented as average ± SD. Significance between treatments (P < 0.05) are indicated by an asterisk (*).

During the treatment phase, drought treatment led to an increase in free amino acid concentration in many amino acids compared to control (**Figure 4**). For example, serine (SER) increased 3310%, aspartic acid (ASP) increased 1851%, and asparagine (ASN) increased 2809% in drought-induced exudates compared to exudates collected from control plants. Additionally, glutamic acid (GLU), tryptophan (TRP), glutamine (GLN), phenylalanine (PHE), and lysine (LYS) were all greater in exudates collected from drought-induced plants (**Figure 4A**). During the recovery phase, ASN and proline (PRO) were increased in root exudates from control plants compared to drought treated plants (**Figure 4B**).

Metabolomic analysis identified a total of 33,870 unique peaks in all 90 samples using FT-ICR-MS. Following filtering, quality control including removal of peaks detected in sampling solution blanks, and molecular formula assignment, 3,985 molecules were detected with 1,616 containing nitrogen in their molecular formula (referred to as N-containing molecules). All molecules were summed in all root exudate samples for a total of 43,413 molecules with 13,142 being N-containing molecules, comprised of 875,625 C and 21,602 N (**Table 4**). All molecules contained between 5-56 C and 0-3 N in their molecular formula.

**Table 4:** All N and C molecules present, their associated count of elements (C or N), and FT-ICR-MS derived C:N ratio of root exudates grouped by experimental phase and treatment.

Experimental phase	Count of molecules		Count of elements		C:N
*Baseline*	N	C	N	C	
Control	383	962	652	19,068	25.1
Drought	417	1,017	696	20,804	25.6
Treatment					
Control	3,710	13,001	6,232	262,350	36.1
Drought	4,193	12,575	6,924	247,068	30.6
Recovery					
Control	2,348	8,363	3,703	170,329	39.4
Drought	2,091	7,495	3,395	156,006	39.4
Total					
	13,142	43,413	21,602	875,625	34.8

As organic products, N-containing molecules contain C and are included in counts of C for all molecules. However, not all molecules include N. The mass C:N ratio uses counts of elements multiplied by their atomic mass (C = 12.011 amu; N = 14.007 amu) to obtain their relative mass within FT-ICR-MS detection range (200 – 900 amu).

**Figure 5:**
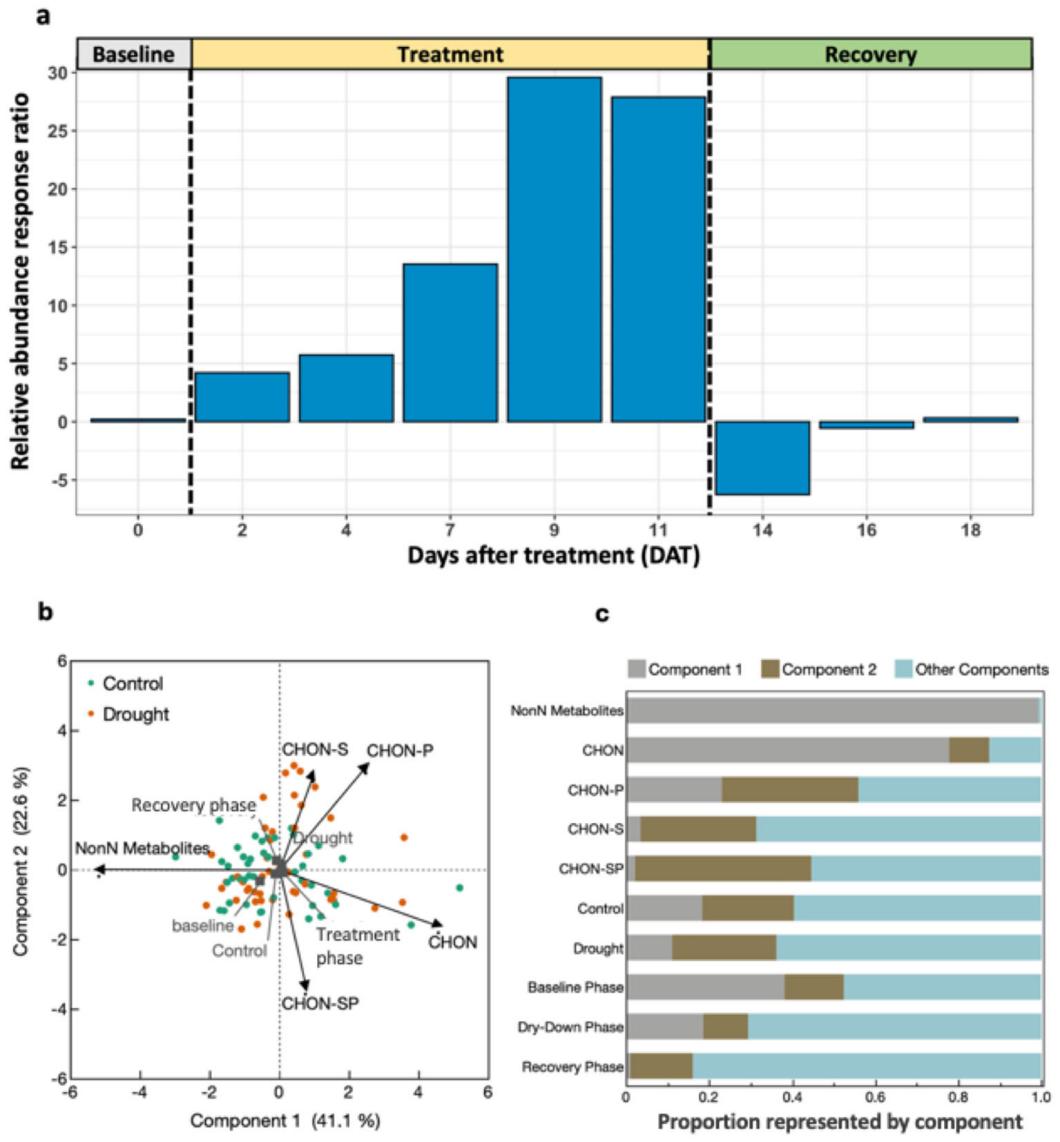
Nitrogen metabolome of root exudates throughout progressive drought and recovery. **(A)** Relative abundance response ratio of N-containing molecule counts. Positive values indicate drought treatment contained higher relative abundance of N-containing molecules than control, averaged by treatment and day. **(B)** Principal components analysis (PCA) of FT-ICR-MS molecules by their elemental composition. **(C)** Squared cosines of PCA factors and supplemental variables across components 1, 2 and components 3-5 (i.e., *Other Components*).

There was an effect of drought (*P* = 0.043) and collection date (*P* = 0.003) on the presence or absence of N-containing molecules in root exudates during the treatment phase, but not baseline or recovery (**Table 5**). The relative abundance response ratio indicated a gradual rise in the enrichment of nitrogen in root exudates as drought stress intensified during the treatment phase (**Figure 5A**). Subsequently, the response ratio rapidly decreased and returned to similar quantities as the control treatment by the end of the experiment when plants had visually fully recovered (**Figure 5B**). A discrepancy between the response ratio becoming similar between treatments by the end of the recovery phase while TON was greater in drought treatment indicates N molecules < 200 or > 900 amu are responsible for increased TON. A PCA of the proportion of N-containing elemental groups (CHON, CHON-S, CHON-P, CHON-SP, etc.) showed that a small majority (63.7%) of variation in the data could be displayed by two axes with the first component representing 41.1% and component two representing 22.6% of variation in data (**Figure 5B**). Metabolites without N (i.e., non-N metabolites) and CHON-composed metabolites were 99% and 78% represented along component one, respectively. Of the supplemental experimental variables included in the PCA, exudates collected during the baseline phase were also primarily represented along component one (**Figure 5C**). Metabolites containing CHON-P (33%) and CHON-SP (42%) were most represented along component two. While the squared cosines for exudates collected from control and drought-treated plants were best represented by component four (shown as other components), loading and factor scores for drought clustered with CHON-S and CHON-P, whereas control and baseline scores clustered with non-N metabolites (**Figures 5B, C**). The molecular mass C:N ratio decreased as an effect of drought during the treatment phase but was consistent between treatments during baseline and recovery phases (**Table 5**). The molecular mass C:N ratio also gradually increased in control treatment as the plants aged, indicating that as the plants grow older or enter reproductive state the root exudate contribution of larger molecules to TON diminishes. While roots released more N as an effect of drought as indicated by TOC/TON (from flow combustion) and molecular mass C:N ratios (from FT-ICR-MS), the C:N ratio of molecular masses is greater than that of TOC/TON. Disparities between C:N ratio determinations are due to the limited detection range of FT-ICR-MS (200-900 m/z) compared with loss on combustion methods, such that N- and C-containing molecules may exist outside the scanning range of FT-ICR-MS. Apart from amino acids, molecules incapable of being detected by FT-ICR-MS were unable to be characterized given the confines of the study.

**Table 5:** Permutational multivariate analysis of variance (PERMANOVA) of the N metabolome of root exudates.

Baseline (day 0)					
	*df*	*Sum of Squares*	*r^2^*	*F*	*P-value*
Treatment	1	0.25	0.08	0.72	0.719
Residual	8	2.81	0.92		
Total	9	3.06	1.00		
Treatment phase (days 2 to 11)					
Treatment	1	0.54	0.03	1.79	**0.043**
Day	4	2.21	0.14	1.83	**0.003**
Treatment × Day	4	1.38	0.09	1.14	0.224
Residual	40	12.10	0.75		
Total	49	16.24	1.00		
Recovery phase (days 14 to 18)					
Treatment	1	0.14	0.02	0.66	0.635
Day	2	0.42	0.07	0.99	0.437
Treatment × Day	2	0.47	0.08	1.11	0.349
Residual	24	5.09	0.83		
Total	29	6.12	1.00		

Three matrices were created using N-containing molecules on a presence or absence basis with Bray-Curtis dissimilarity. Significance is determined at α = 0.05.

Bold values indicate significance (α = 0.05) between treatments.

**Figure 6:**
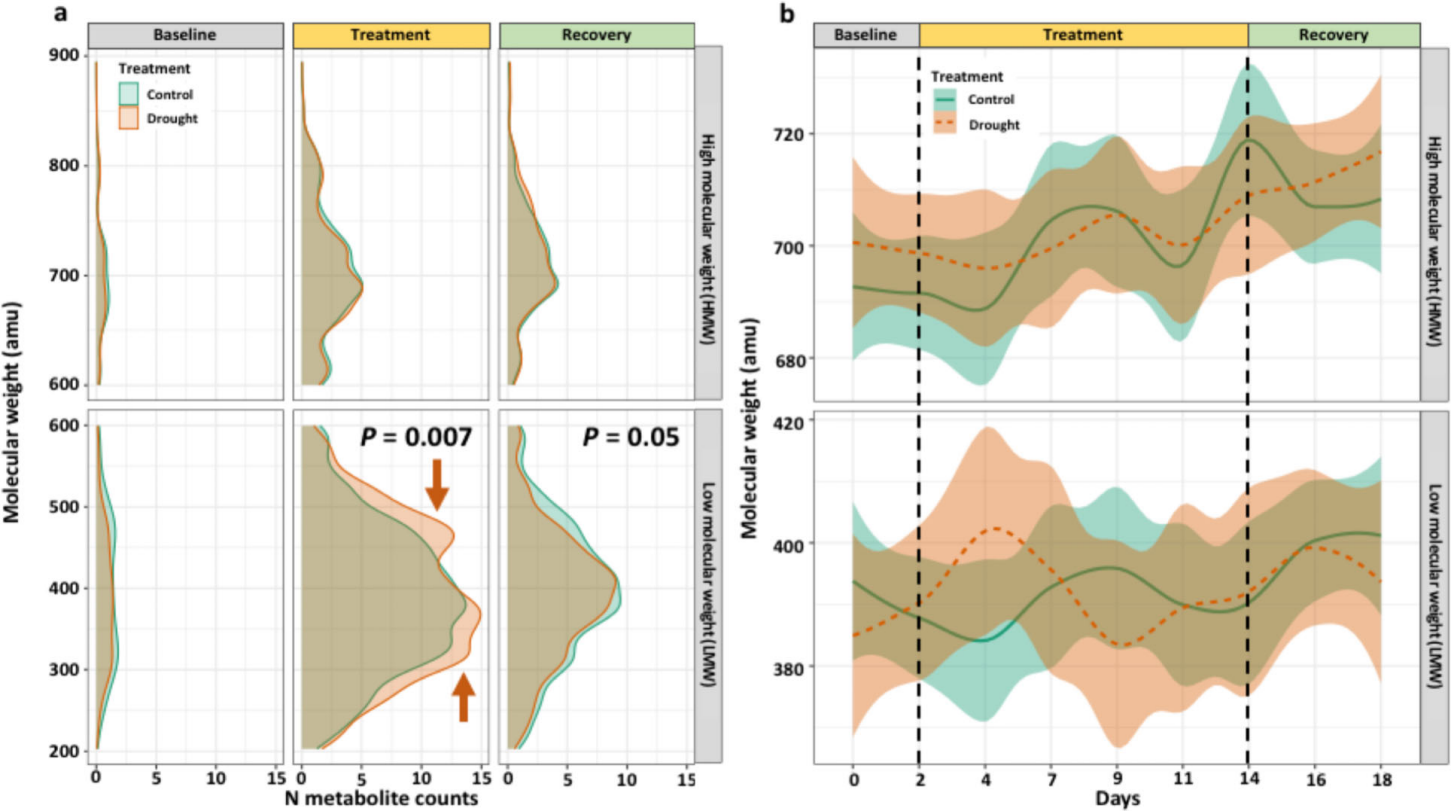
Distributions of the molecular weight for all N-containing molecules split into experimental phases, plotted as **(A)** smoothed density of N molecule counts and **(B)** LOESS regression with 50% spline of average molecular weight for treatments by day. Distributions differ during the treatment and recovery phases for LMW molecules, with drought-induced regions indicated with orange arrows. Statistical tests were Kolmogorov-Smirnov tests of distribution (α = 0.05) between treatments at that sampling timepoint.

The molecular masses of N-containing molecules were predominantly LMW (i.e., 200-600 amu) with only 25-30% being HMW (600-900 amu). Drought affected the distribution of LMW molecules during the treatment (*P* = 0.007) and recovery phases (*P* = 0.05) (**Figure 6A**). During the treatment phase, two induced LMW regions were identified from 275 – 390 and 450 – 550 amu in drought treatment (**Figure 6A**). However, these two peaks were not present during the recovery phase (**Figure 6A**). The average molecular weight of N-containing molecules over time contained a large degree of variability and did not differ among LMW or HMW molecules (**Figure 6B**).

### Drought-induced unique molecules

**Figure 7:**
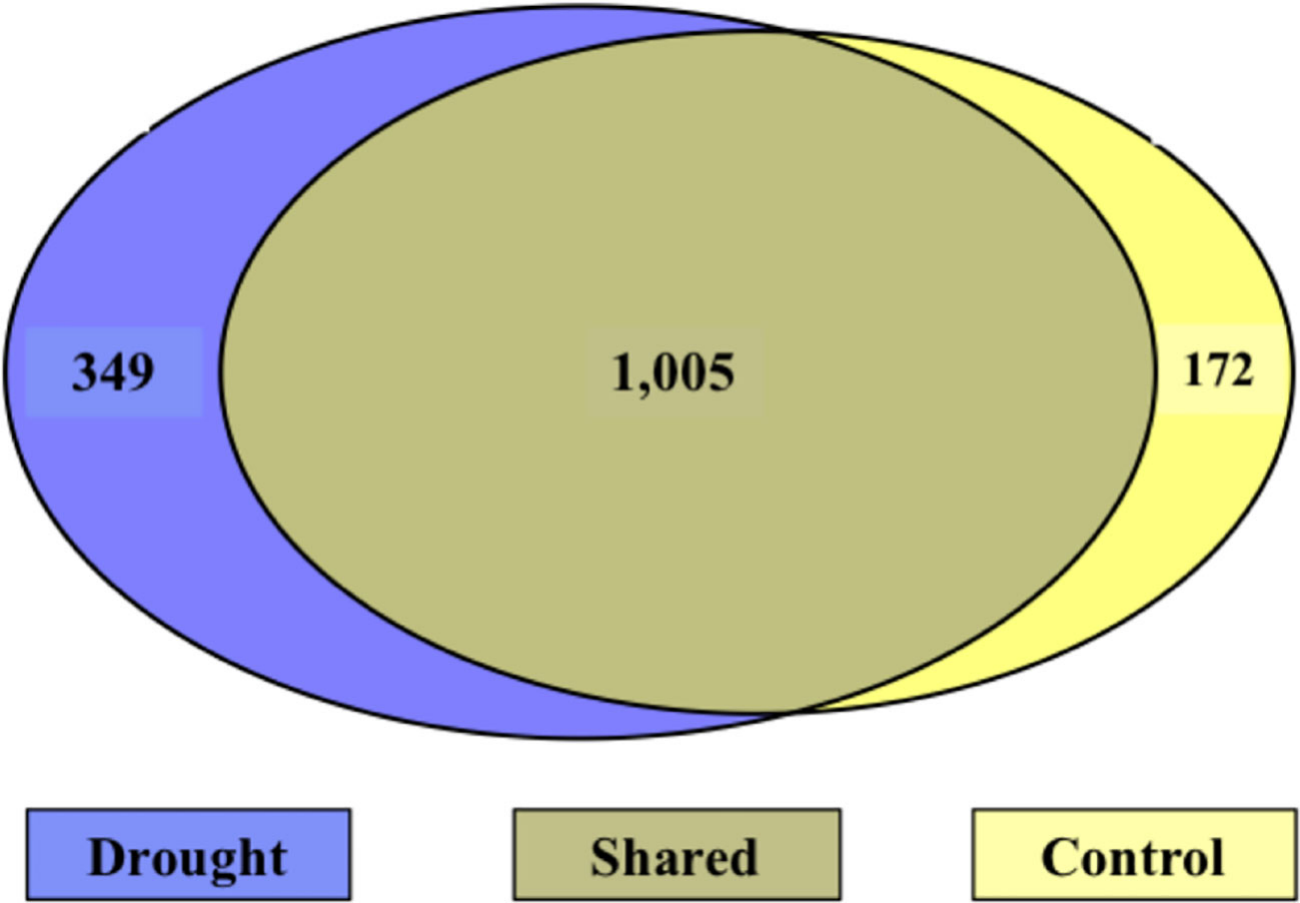
Venn diagram of N-containing molecules during the treatment phase. Left (blue) is unique to drought, right (yellow) is unique to control, and the center is shared. The majority of molecules were shared between treatments.

During the treatment phase, there were 349 N-containing molecules unique to drought, 172 unique to control, and 1,005 shared between treatments (**Figure 7**). Of the N-containing molecules unique to drought treatment, 10 N-containing molecules were selected as important to the drought response based on a presence in at least 3 days (out of 5) and detection at least 5 times (**Table 6**). Interestingly, the masses of the 10 selected molecules all occurred in the drought-induced LMW regions (275 – 390 and 450 – 550 amu) identified in **Figure 4A**, while 65% of the 349 unique drought N-containing molecules occurred there.

**Table 6:** Ten N-containing molecules unique to drought during the treatment phase were selected for their importance to the drought response of plants.

Mass (amu)	Molecular formula	Days present	Occurrence
266.15	C_13_H_21_O_3_N_3_	3	9
308.08	C_14_H_15_O_7_N	4	7
336.33	C_22_H_43_ON	3	6
337.20	C_15_H_34_O_2_N_2_S_2_	3	10
340.20	C_18_H_32_O_3_NP	3	7
360.24	C_18_H_35_O_6_N	3	11
370.13	C_21_H_25_ONS_2_	3	22
384.15	C_22_H_27_ONS_2_	3	22
455.12	C_33_H_16_ON_2_	3	5
473.28	C_30_H_38_O_3_N_2_	3	5
489.28	C_30_H_38_O_4_N_2_	3	6

## Discussion

In this experiment, time-series progressive drought revealed that root exudation of organic N was substantially increased and sensitive to drought stress and recovery. Organic molecules comprised a substantial portion of root exudate N with amino acids and NO_3_^-^ being < 5% of root exudate total N. Root exudate TON represented 20-35% of TOC, which was consistent with estimation by FT-ICR-MS that 25-40% of the molecules contained N. Being that the overall prevalence of LMW N containing molecules was greater than HMW, and C:N ratio obtained from flow combustion were reduced in drought treatment during the recovery phase, it is suspected that the origin of the increased N derived from molecules < 200 amu that were not quantified to be amino acids. Interestingly, no N-containing molecules were identified by KEGG libraries (although the same dataset yielded high level insights to overall metabolome, e.g. Lin et al., 2023), which highlights both the complexity of the root exudate N metabolome and a current lack of database coverage for these N molecules. When the root exudate metabolome was analyzed with PCA, the N-containing molecules grouped separately depending upon their elemental composition, similar to other FT-ICR-MS observations of root exudates during drought (Ulrich et al., 2022). These data align with studies on drought modifications to N-containing molecules, such as amino acids, proteins, amino sugars, and CHON metabolites in other species (Jiang et al., 2023; Bobille et al., 2019; Calvo et al., 2017; Preece et al., 2023; Canarini et al., 2016; Gargallo-Garriga et al., 2018; Ghatak et al., 2022; Tiziani et al., 2022) and advance the literature to further suggest that changes to the quantity and quality of organic N in root exudates are a sensitive and underrepresented strategy by which root exudates respond to drought stress.

Amino acids and small peptides (< 500 amu) are considered prominent N sources of root exudates (Okumoto and Pilot, 2011). The most abundant free amino acids observed to be exuded by cotton (e.g., aspartic acid, glycine, serine, tyrosine, glutamine, threonine, glutamic acid) were similar to those amino acids commonly identified in high concentrations across a wide variety of soils (Lipson and Näsholm, 2001). A study in white clover identified glycine, serine, and alanine as the major amino acids in root exudates (Lesuffleur et al., 2007). Gargallo-Garriga et al. (2018) reported in holm oak that drought increased the concentrations of phenylalanine, tyrosine, and tryptophan, which are precursors for the synthesis of alkaloids and terpenoids. In a study of three evolutionary stages of wheat, the modern variety was shown to exude higher concentrations of tryptophan, histidine, and phenylalanine compared to the wild-type modern varieties (Shaposhnikov et al., 2016). Tryptophan has been foliar applied to wheat in tandem with *Pseudomonas fluorescens* and proven to induce drought tolerance in an experimental pot study (Jamil et al., 2018). Calvo et al. (2017) found no differences in free amino acids during water stress in barley. Drought stress can lead to changes in the osmolyte composition of root tissues, potentially increasing the release of amino acids. Overall, the magnitude amino acid-N increased drastically (562%) as a result of the drought treatment but contributed very little to TON.

Surprisingly, no N-containing molecules identified by FT-ICR-MS were identified in KEGG databases. A lack of molecule annotation could be attributed to multiple factors such as limited database coverage, molecule complexity and fractionation during processing, post-translational modifications, database updates, and data availability, particularly for specialized or less common compounds. However, the same FT-ICR-MS data using all molecules was used to identify 68 pathways and more than 17 modules by Lin et al. (2023) in a broad coverage analysis, identifying such pathways as the biosynthesis of flavonoid compounds corresponding to progressive drought. It is clear that N-containing molecules are prevalently associated with metabolic pathways that are difficult to annotate, have limited database coverage, and may be a result of previously taken up nutrient N that was bound and exuded in organic forms.

Root exudation of nutrient “poor” carbon compounds induces microbes to degrade existing soil compounds that contain N (Craine et al., 2007), so that the acceleration of the C cycle is primed by exogenous C input and controlled by N supply (Chen et al., 2022). The lower C:N ratio during drought observed in root exudates is in line with findings from temperate grasses (Li et al., 2021) and tomato (Preece et al., 2023), while increased TOC root exudation aligns with studies on drought induced root exudation (Chen et al., 2022; Preece et al., 2018). Progressive drought also led to an increased distribution of LMW N-containing molecules, but not HMW, with molecules containing 1 N having the largest increase over molecules with 2 and 3 N in drought. Waldo et al. (2021) observed in a peatland bog that microbial priming of root exudates triggered increased processing of high molecular weight molecules regardless of molecular content but only processed low molecular weight (< 500 amu) compounds if they contained N or S. Thus, the assimilatory coupling of N and C cycles through microbial metabolism (Burgin et al., 2011) presents a complex interaction that may differ between environments and plant species as N exudation varies in response to drought and other edaphic factors.

The aeroponic system mimics nutrient availability in the soil since nutrient delivery to the plant is regulated by water diffusion to its roots. When water is limited, nutrient fluxes to the root are limited correspondingly to the water flux. Thus, during drought treatment, plants had lower N availability. Despite this, organic N exudation increased during drought in plants receiving overall less N. He et al. (2022) observed in cotton roots that water-stress reduced N absorption but increased concentration of photosynthates, ultimately reducing leaf photosynthetic N utilization. It is important to note that in our study, the Hoagland’s nutrient solution that was used constitutes a high fertility environment as nutrients are soluble and readily accessible to the plant. Thus, it may be economically strategic for a droughted plant to exude more N if it can leverage some benefit by doing so in a high fertility environment, as shown by wheat which exuded more N in a high fertility environment (Janzen, 1990). It is unknown whether this strategy would occur in oligotrophic environments. Further studies using varying N regimes would benefit to answer these questions and better account for diverse fertility landscapes exposed to water-limiting conditions.

A potential mechanism by which plants respond to drought is the alteration of root exudates that leads to controlled microbial respiration and the end product of water (de Vries et al., 2019), with evidence of enhanced N exudation being linked to greater microbial activity when C supplies are limited (Waldo et al., 2021). By suppling C- and N-rich exudates to microorganisms, the plant is benefited by microbial production of biofilms that surround the rhizoplane and maintain root hydration (Wang et al., 2019; Timmusk et al., 2015). Additionally, drought increased root exudation of organic N may help to maintain N mineralization rates since the aminization of organic N leads to end products such as CO_2_, NH_4_, SO_4_, and importantly water. Because plant investment into aboveground growth is halted during drought, the cost of releasing organic N into the rhizosphere may come as a reasonable tradeoff if root tip hydration is modulated by enhanced microbial activity in a drying soil environment.

These findings are the first time-series experiment to highlight that drought-induced changes to TON root exudation are more substantial than TOC exudation. While the current study is limited in that results are from a soilless system, there are few alternatives to collect root exudates in time-series analysis from the same plants overtime; thus, future studies should attempt to build upon these results in soil systems. From here there is an exciting path for linking C and N exudation to microbial assembly in the rhizosphere, engineering drought resilient rhizospheres, and challenging traditional models of N biogeochemistry given the substantial allocation to root exudation of organic N during low water availability.

## Data Availability

The raw data supporting the conclusions of this article will be made available by the authors, without undue reservation.
